# Cytotoxic and Anti-Inflammatory Triterpenoids in the Vines and Leaves of *Momordica charantia*

**DOI:** 10.3390/ijms23031071

**Published:** 2022-01-19

**Authors:** Mei-Chia Chou, Yuan-Jia Lee, Yao-Ting Wang, Shi-Yie Cheng, Hsueh-Ling Cheng

**Affiliations:** 1 Graduate Institute of Bioresources, National Pingtung University of Science and Technology, Neipu, Pingtung County 912301, Taiwan; meeichia@gmail.com; 2 Department of Physical Medicine and Rehabilitation, Kaohsiung Veterans General Hospital, Pingtung Branch, Neipu, Pingtung County 912012, Taiwan; 3 Department of Recreation and Sports Management, Tajen University, Yanpu, Pingtung County 907101, Taiwan; 4 Department of Physical Therapy, Shu-Zen Junior College of Medicine and Management, Kaohsiung 82144, Taiwan; 5 Department of Biological Science and Technology, National Pingtung University of Science and Technology, Neipu, Pingtung County 912301, Taiwan; asd2691276@yahoo.com.tw; 6 Department of Life Sciences, National University of Kaohsiung, Kaohsiung 811726, Taiwan; x19931027@gmail.com

**Keywords:** *Momordica charantia*, triterpenoid, anti-inflammation, antioxidant enzymes, Nrf2, heme oxygenase-1, cytotoxicity

## Abstract

The vines and leaves of *Momordica charantia* L. are used as herbal medicines to treat inflammation-related disorders. However, their safety profile remains uncharacterized, and the constituents in their extracts that exert anti-inflammatory and adverse effects remain unclear. This study isolated the characteristic cucurbitane-type triterpenoid species in the vines and leaves of *M. charantia* L. and analyzed their cytotoxicity, anti-inflammatory effects, and underlying mechanisms. Four structurally related triterpenoids—momordicines I, II, IV, and (23E) 3β,7β,25-trihydroxycucurbita-5,23-dien-19-al (TCD)—were isolated from the triterpenoid-rich fractions of extracts from the vines and leaves of *M. charantia*. Momordicine I was cytotoxic on normal cells, momordicine II exerted milder cytotoxicity, and momordicine IV and TCD had no obvious adverse effects on cell growth. TCD had anti-inflammatory activity both in vivo and in vitro. In lipopolysaccharide-stimulated RAW 264.7 cells, TCD inhibited the inhibitor kappa B kinase/nuclear factor-κB pathway and enhanced the expression of nuclear factor erythroid 2-related factor 2, heme oxygenase-1, and glutamate-cysteine ligase modifier subunit through the extracellular signal-regulated kinase1/2 and p38. Thus, the vines and leaves of *M. charantia* should be used with caution. An extraction protocol that can enrich TCD but remove momordicine I would likely enhance the safety of the extract.

## 1. Introduction

*Momordica charantia* L. belongs to the Cucurbitaceae family, and its fruit is commonly known as bitter melon or bitter gourd. Each part of this plant, including the vines, leaves, fruits, roots, and seeds, has been used in folk medicine to treat various diseases, including diabetes, worm infection, and inflammation [1,2]. However, under some circumstances, *M. charantia* L. has also exerted adverse effects on human health. Therefore, its safety requires comprehensive evaluation [1,2].

Chronic inflammation is associated with the development of diseases including metabolic syndrome, type 2 diabetes, cardiovascular diseases, cancer, and neurodegenerative diseases [3]. Aside from the fruits, the vines and leaves of *M. charantia* L. are also used as herbal medicines in treating inflammation-related disorders [1,2]. However, unlike the fruits, they are not commonly consumed as food; thus, their safety profile remains uncharacterized. Moreover, the components of their extracts that exert anti-inflammatory effects and adverse effects are unclear. The vines and leaves of *M. charantia* L. are rich in cucurbitane-type triterpenoids [4,5], which possess 19-(10→9*β*)-*abeo*-10*α*-lanost-5-ene skeletons and were originally discovered from the *Momordica* species [6]. The safety, anti-inflammatory activities and mechanisms of these compounds are not fully addressed. In this study, we isolated characteristic cucurbitane-type triterpenoid species in the vines and leaves of *M. charantia* L. and analyzed their cytotoxicity, anti-inflammatory effects, and underlying mechanisms.

## 2. Results

### 2.1. Cucurbitane-Type Triterpenoids Isolated from the Vines and Leaves of M. charantia

**Figure 1:**
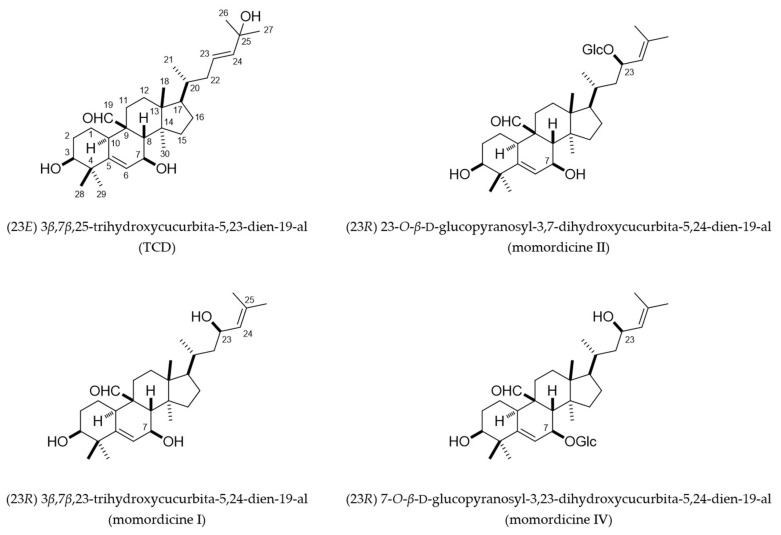
Chemical structures of (23E) 3β,7β,25-trihydroxycucurbita-5,23-dien-19-al (TCD) and momordicines I, II, and IV. Glc, glucose.

**Figure 2:**
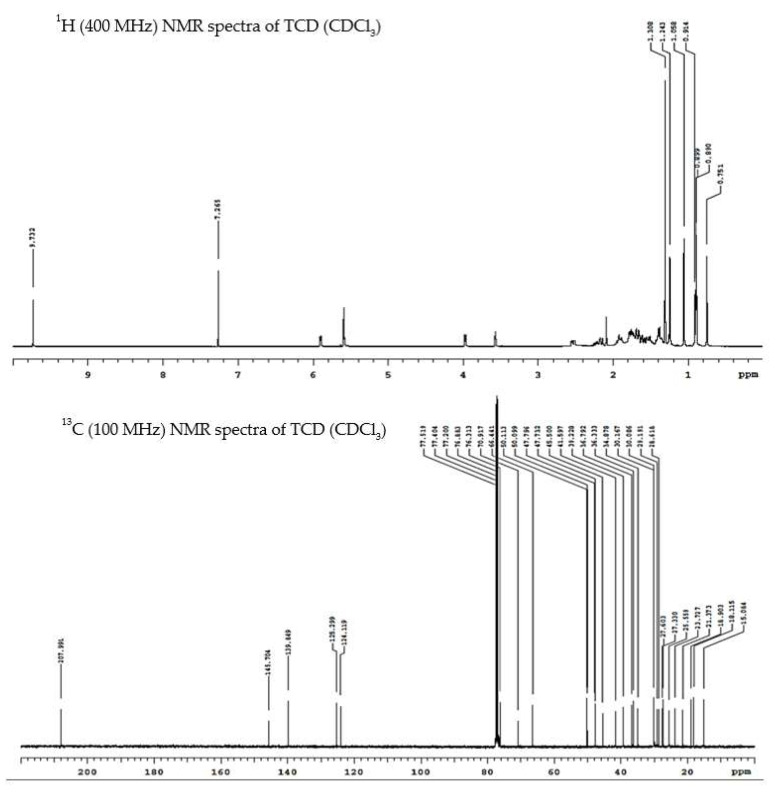
^1^H and ^13^C nuclear magnetic resonance (NMR) spectroscopy data of TCD.

From the triterpenoid-rich fractions of the vines and leaves of *M. charantia* (see Section 4.4), we isolated four previously characterized metabolites: (23E) 3β,7β,25-trihydroxycucurbita-5,23-dien-19-al (TCD), 3β,7β,23-trihydroxycucurbita-5,24-dien-19-al (momordicine I), (23R) 23-*O*-β-d-glucopyranosyl-3,7-dihydroxycucurbita-5,24-dien-19-al (momordicine II), and (23R) 7-*O*-β-d-glucopyranosyl-3,23-dihydroxycucurbita-5,24-dien-19-al (momordicine IV) (Figure 1). The structures of these metabolites were elucidated by comparing their ^1^H and ^13^C nuclear magnetic resonance (NMR) spectroscopy data (Figure 2 and Supplementary Materials) with those of related known compounds [7,8,9]. TCD and momordicine I are isomers, with the –OH group on C-25 and the double bond between C-23 and C-24 in TCD being shifted in momordicine I. Momordicines II and IV are glucosides of momordicine I. Presumably, these isolates are likely important components underlying the biological activities of the extract prepared from the title plant because of their relative high abundance in the extract (2.23% of the total weight of the EtOAc extract for TCD, 3.03% for momordicine I, 0.22% for momordicine II, and 0.21% for momordicine IV; see Section 4.4).

To compare the lipophilicity of the compounds, the partition coefficient logP was calculated for each compound. Their logP values from high (more lipophilic) to low (less lipophilic) are TCD 5.17; momordicine I 4.41; momordicine II 2.72; and momordicine IV 2.72 (Supplementary Materials).

### 2.2. Cytotoxicity of Triterpenoids

**Figure 3:**
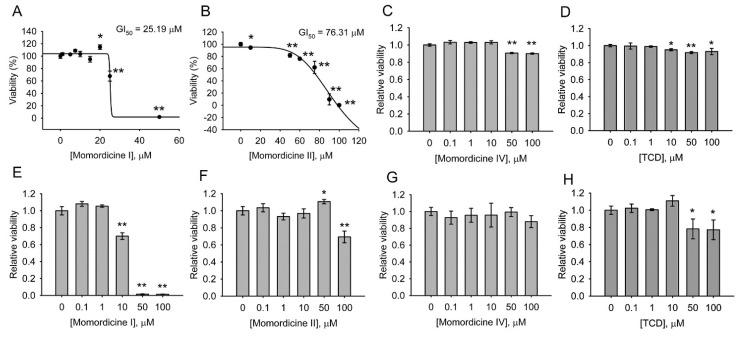
Cytotoxic analysis of momordicines I, II, and IV and TCD in IEC-18 and FL83B cell lines: (**A**–**D**) IEC-18 cells; (**E**–**H**) FL83B cells. The cells were treated with the indicated concentrations of momordicine I (**A**,**E**), momordicine II (**B**,**F**), momordicine IV (**C**,**G**), or TCD (**D**,**H**) for 24 h. Cell survival relative to the control ([compound] = 0 µM) was determined. In (**A**,**B**), the GI_50_ of the compound was calculated. The experiments were performed in triplicate. Data are presented as means ± standard deviations. * *p* < 0.05, ** *p* < 0.005 versus the control.

Momordicines I, II, and IV and TCD were first characterized for cytotoxicity by using a normal intestinal cell line, IEC-18, and a normal hepatic cell line, FL83B. Figure 3A–D indicate that momordicine I significantly inhibited the growth of IEC-18 cells, with a GI_50_ (concentration required for inhibiting cell growth by 50%) of 25.19 µM (A); the toxicity of momordicine II was milder, with a GI_50_ of 76.31 µM to IEC-18 cells (B). By contrast, momordicine IV (C) and TCD (D) maintained over 80% cell survival in the concentration range of 0.1–100 µM. Figure 3E–H demonstrate that momordicine I (E) was lethal to FL83B cells at 50 and 100 µM and slightly but definitively suppressed cell growth at 10 µM. Momordicine II (F), momordicine IV (G), and TCD (H) were less toxic to FL83B cells; under 0.1–100 µM, cell survival exceeded 70%. These data suggest that momordicine I is the most toxic to normal cells among the four compounds, exerting deleterious effects on both cell lines in concentrations higher than 10 or 20 µM. Momordicine II produced a milder detrimental effect on IEC-18 cells. Momordicine IV and TCD were not harmful to normal cells in the indicated concentrations.

**Figure 4:**
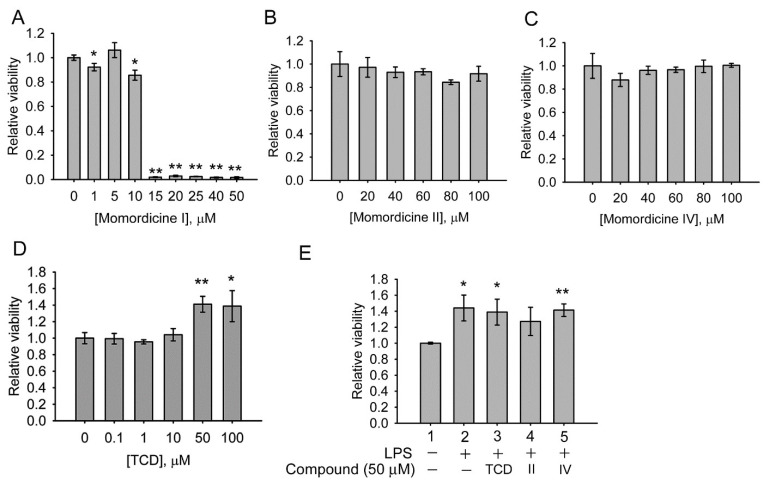
Cytotoxic analysis of the effects of momordicines I, II, and IV and TCD on RAW 264.7 cells. (**A**–**D**) The cells were treated with the indicated concentrations of momordicine I (**A**), momordicine II (**B**), momordicine IV (**C**), or TCD (**D**) for 24 h. (**E**) The cells were cotreated with 100 ng/mL lipopolysaccharides (LPS) and 50 µM TCD (Group 3), momordicine II (Group 4), momordicine IV (Group 5), LPS alone (Group 2), or the solvent (Group 1) for 24 h. Cell survival relative to the control ([compound] = 0 µM or Group 1) was determined. The experiments were performed in triplicate. Data are presented as means ± standard deviations. * *p* < 0.05, ** *p* < 0.005 versus the control.

The cytotoxicity of the compounds on the model macrophage cell line RAW 264.7 was examined. Momordicine I significantly inhibited cell survival at concentrations of ≥15 µM (Figure 4A). Momordicine II (Figure 4B), momordicine IV (Figure 4C), and TCD (Figure 4D) were not toxic to RAW 264.7 cells at 20–100 or 0.1–100 µM, and TCD at 50–100 µM actually increased cell growth. Next, the effects of these three compounds on cell survival in the presence of lipopolysaccharides (LPS) were examined. As presented in Figure 4E, LPS cotreatment with 50 µM TCD (Group 3), momordicine II (Group 4), or momordicine IV (Group 5) was not toxic to RAW 264.7 cells. The results indicate that only momordicine I is toxic to RAW 264.7 cells.

### 2.3. Anti-Inflammatory Effects of Triterpenoids

**Figure 5:**
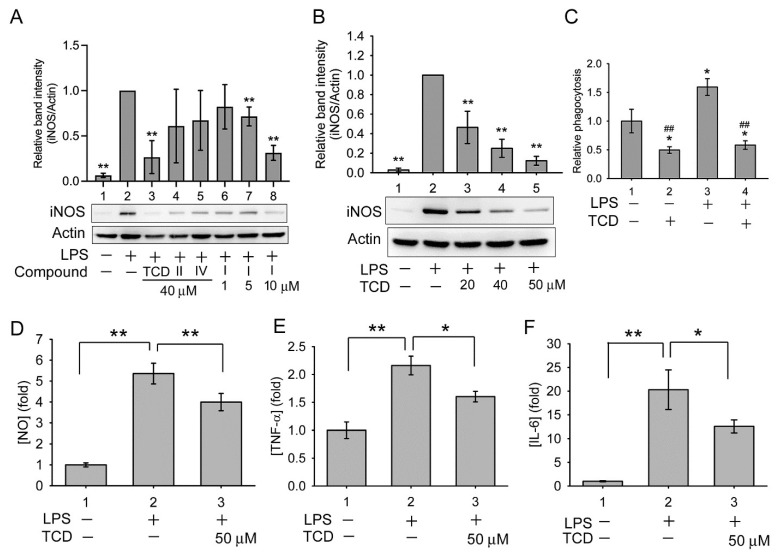
TCD reduced the expression of LPS-induced inflammatory biomarkers in RAW 264.7 cells. (**A**,**B**) Western blotting analysis of inducible nitric oxide synthase (iNOS) expression: (**A**) cells were stimulated with 100 ng/mL LPS (Lanes 2–8) and cotreated with 40 µM TCD (Lane 3), momordicine II (Lane 4), or momordicine IV (Lane 5) for 16 h or with 1, 5, or 10 µM momordicine I (Lanes 6, 7, and 8, respectively) for 16 h; (**B**) cells were treated with LPS and 20, 40, or 50 µM TCD (Lanes 3, 4, and 5, respectively), or LPS alone (Lane 2) for 16 h. Relative band intensity (normalized by actin) versus Lane 2 was determined. Data are presented as the means ± standard deviations of four (**A**) or three (**B**) independent experiments. * *p* < 0.05 and ** *p* < 0.005 versus Lane 2. (**C**) Cells were stimulated with solvent (Group 1), 50 µM TCD (Group 2), 100 ng/mL LPS (Group 3), or cotreated with LPS and TCD (Group 4) for 24 h. This was followed by a phagocytosis assay. Phagocytic activity relative to that of Group 1 was determined. Data are presented as the means ± standard deviations of experiments performed in triplicate. * *p* < 0.05 versus Group 1; ## *p* < 0.005 versus Group 3. (**D**–**F**) Cells were stimulated with the solvent (Group 1) or 100 ng/mL LPS (Group 2) or cotreated with LPS and 50 µM TCD (Group 3) for 12 h (**E**) or 16 h (**D**,**F**), and the medium was subjected to [NO] (**D**), tumor necrosis factor α ([TNF-α]) (**E**), or interleukin-6 ([IL-6]) (**F**) analysis. Data are presented as means ± standard deviations (N = 9 in (**D**); N = 3 in (**E**) and (**F**)). * *p* < 0.05, ** *p* < 0.005 between the indicated groups.

The anti-inflammatory effects of momordicine II, momordicine IV, and TCD were compared in LPS-treated RAW 264.7 cells. As presented in Figure 5A, LPS increased the expression of inducible nitric oxide synthase (iNOS) in the cells (Lane 2 vs. Lane 1), whereas the addition of 40 µM TCD (Lane 3) significantly suppressed iNOS expression compared with LPS treatment alone (Lane 2). By contrast, momordicine II (Lane 4) or momordicine IV (Lane 5) did not significantly inhibit iNOS expression. Although momordicine I is toxic to RAW 264.7 cells, we evaluated its anti-inflammatory effect using sublethal concentrations. As displayed in Figure 5A (Lanes 6–8), 1–10 µM momordicine I dose-dependently inhibited iNOS expression, implying that it has anti-inflammatory activity at sublethal concentrations.

Overall, the results suggest that TCD and momordicine I both possess anti-inflammatory activities. However, momordicine I is cytotoxic. Therefore, we further characterized the anti-inflammatory effects of TCD and the underlying mechanisms.

As presented in Figure 5B, 20–50 µM TCD dose-dependently inhibited LPS-induced iNOS expression. Moreover, compared with the control, LPS increased the phagocytic activity of RAW 264.7 cells (Group 3), whereas TCD alone (Group 2) or with LPS (Group 4) significantly reduced the phagocytic activity (Figure 5C). Furthermore, LPS triggered NO production in RAW 264.7 cells (Figure 5D, Group 2), and TCD partially but significantly inhibited LPS activity (Figure 5D, Group 3). Similarly, LPS increased the expression of tumor necrosis factor α (TNF-α) (Figure 5E, Group 2) and interleukin-6 (IL-6) (Figure 5F, Group 2), both of which were significantly suppressed by the addition of TCD (Figure 5E,F, Group 3). These results demonstrate that TCD possesses anti-inflammatory activity. 

**Figure 6:**
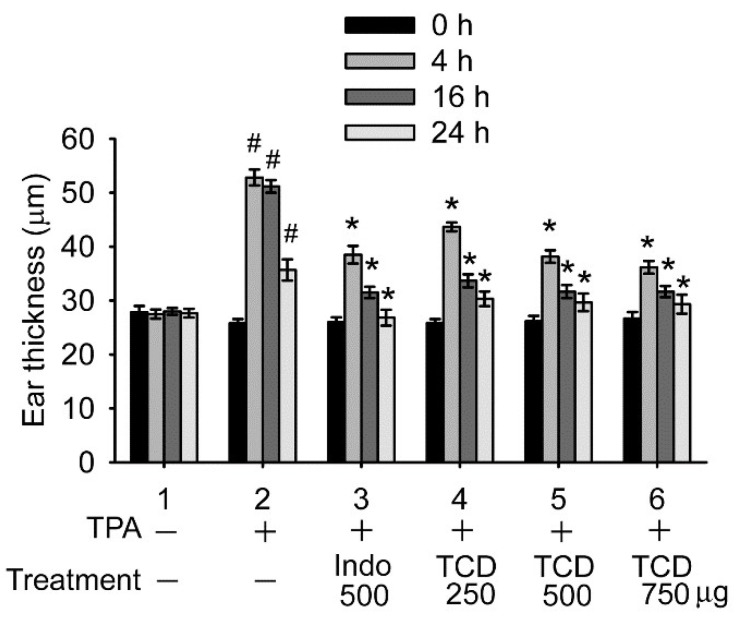
TCD ameliorated 12-O-tetradecanoylphorbol-13-acetate (TPA)-induced ear edema in vivo. Mice were treated with TPA (Groups 2–6) or the solvent (Group 1) in one ear. One hour later, solvent (Groups 1 and 2), 500 μg/ear indomethacin (Group 3), or 250, 500, or 750 μg/ear TCD (Groups 4, 5, and 6, respectively) was applied on the ear. Ear thickness was measured before (0 h) and at 4, 16, and 24 h after TPA treatment. Data are presented as the means ± standard deviations of each group (N = 6). # *p* < 0.001 versus 0 h of Group 2; * *p* < 0.001 versus the same time point of Group 2.

As shown in Figure 6, 12-*O*-tetradecanoylphorbol-13-acetate (TPA) caused ear edema, a sign of ear inflammation, in treated mice 4, 16, and 24 h after TPA stimulation (Group 2). TCD treatment ameliorated ear edema. All tested doses (250, 500, and 750 µg/ear in Groups 4, 5, and 6, respectively) of TCD significantly reduced the degree of ear edema in mice 4, 16, and 24 h after TPA stimulation compared with the TPA control (Group 2). The effects of 500 and 750 µg/ear TCD on ear inflammation reduction were comparable to that of 500 µg/ear indomethacin, a nonsteroidal anti-inflammatory drug (Group 3). These results confirm that TCD can ameliorate inflammation in vivo.

### 2.4. Anti-Inflammatory Mechanism of TCD

**Figure 7:**
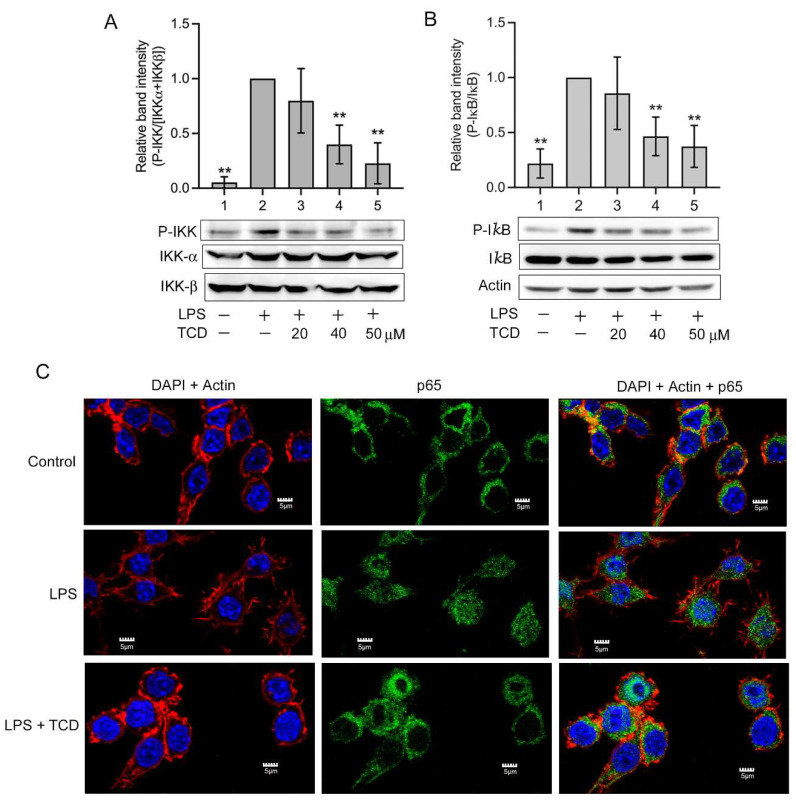
TCD inhibited the inhibitor kappa B kinase (IKK)/nuclear factor-κB (NF-κB) pathway. (**A**,**B**) Western blot analysis. RAW 264.7 cells were treated with the solvent (Lane 1) or 100 ng/mL LPS (Lane 2), or co-treated with LPS and 20, 40, or 50 μM TCD (Lanes 3, 4, and 5, respectively) for 1 h. The levels of IKK phosphorylation (**A**) and the inhibitor of NF-κB (IκB) phosphorylation (**B**) were analyzed. Normalized relative band intensity versus Lane 2 was determined. Data are presented as the means ± standard deviations of four independent experiments. ** *p* < 0.005 versus Lane 2. (**C**) Confocal microscopy analysis. RAW 264.7 cells were treated with the solvent (control), 100 ng/mL LPS alone (LPS), or LPS and 50 μM TCD (LPS + TCD) for 1 h. Cells were labeled with a p65-specific antibody and an FITC-conjugated secondary antibody (green fluorescence), rhodamine phalloidin (actin labeling for locating the cell membrane; red fluorescence), and 4′,6-diamidino-2-phenylindole (DAPI; nuclear staining; blue fluorescence). The superimposed images of DAPI staining and rhodamine phalloidin staining (DAPI + Actin), the results of FITC labeling (p65), and the superimposed images of all staining (DAPI + Actin + p65) are shown. Scale bars: 5 μm.

LPS activates the inhibitor kappa B kinase (IKK)/nuclear factor-κB (NF-κB) pathway through the activation of toll-like receptor 4 [10,11]. Thus, we determined whether TCD inhibited the IKK/NF-κB pathway. As presented in Figure 7A, LPS caused an apparent increase in phosphorylated IKK (Lane 2) in RAW 264.7 cells. The addition of 20–50 μM TCD (Lanes 3, 4, 5) dose-dependently inhibited LPS-induced IKK phosphorylation. Moreover, Figure 7B demonstrates that LPS increased the phosphorylation of the inhibitor of NF-κB (IκB) (Lane 2), a substrate of IKK, but that cotreatment of 20–50 μM TCD with LPS dose-dependently reduced the level of IκB phosphorylation (Lanes 3–5). Furthermore, the nuclear translocation of NF-κB was examined using confocal microscopy. As presented in Figure 7C, in solvent-treated control cells, the location of the NF-κB subunit p65 was perinuclear, suggesting that NF-κB is located outside the nucleus. In LPS-treated cells, the location of p65 overlapped with the 4′,6-diamidino-2-phenylindole (DAPI)-labeled nucleus, indicating that NF-κB translocated into the nucleus in these cells. Under cotreatment with LPS and TCD, p65 exhibited perinuclear distribution in most cells, suggesting that TCD inhibited the LPS-induced nuclear translocation of NF-κB. Furthermore, the morphologies of RAW 264.7 cells differed among groups. In the control and LPS + TCD groups, cell morphology was similar to that of fibroblasts or endothelial cells. By contrast, in the LPS group, the cells exhibited a macrophage-like morphology, with numerous needle-like protrusions extending from the cell membrane. This suggested that LPS induced the differentiation of RAW 264.7 cells into macrophage-like proinflammatory cells [12,13], yet TCD suppressed the differentiation. Overall, the results imply that TCD suppressed the LPS-activated IKK/NF-κB pathway.

**Figure 8:**
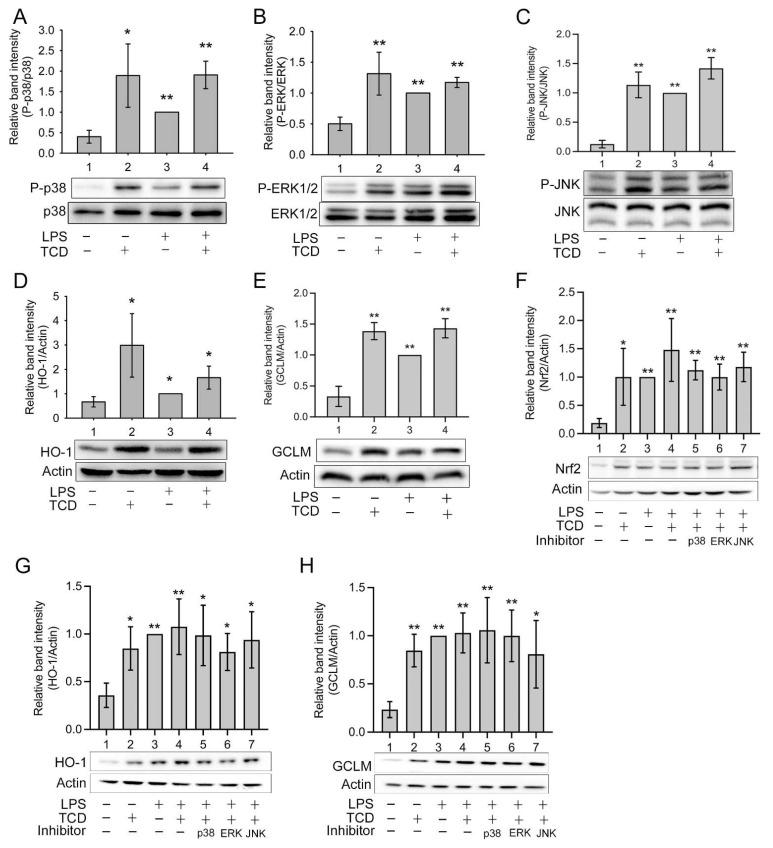
TCD activated mitogen-activated protein kinases (MAPKs) and the nuclear factor erythroid 2-related factor 2 (Nrf2)/heme oxygenase-1 (HO-1) pathway. RAW 264.7 cells were treated with the solvent (Lane 1), 50 μM TCD (Lane 2), 100 ng/mL LPS (Lane 3), or LPS and TCD (Lane 4) for 1 h (**A**–**C**), 24 h (**D**,**E**), 2 h (**F**), or 6 h (**G**,**H**). The levels of phosphorylated p38 (**A**), phosphorylated extracellular signal-regulated kinase1/2 (ERK1/2) (**B**), phosphorylated c-Jun N-terminal kinase (JNK) (**C**), HO-1 (**D**,**G**), glutamate–cysteine ligase modifier subunit (GLCM) (**E**,**H**), and Nrf2 (**F**) were analyzed using Western blotting. In (**F**,**G**,**H**), an inhibitor (20 μM) of p38 (Lane 5), ERK1/2 (Lane 6), or JNK (Lane 7) was also added to cells cotreated with LPS and TCD. Normalized relative band intensity versus Lane 3 was determined. Data are presented as the means ± standard deviations of four independent experiments. * *p* < 0.05, ** *p* < 0.005 versus Lane 1. In (**F**,**G**,**H**), statistical analysis of Lanes 5, 6, and 7 versus Lane 4 was also performed, but no significant difference was found.

In addition to activating IKK, TLR4 also activates mitogen-activated protein kinases (MAPKs) p38, c-Jun-N-terminal kinase (JNK), and extracellular signal-regulated kinase1/2 (ERK1/2), which activate AP-1 and promote the expression of some proinflammatory cytokines [10,11]. Therefore, the effects of LPS and TCD on MAPKs were also explored. Figure 8A–C reveals that TCD (Lane 2), LPS (Lane 3), and LPS + TCD (Lane 4) clearly activated p38 (A), ERK1/2 (B), and JNK (C) compared with the control (Lane 1). The results indicate that TCD and LPS both activated MAPKs and that TCD did not suppress LPS-induced MAPK activation.

**Figure 9:**
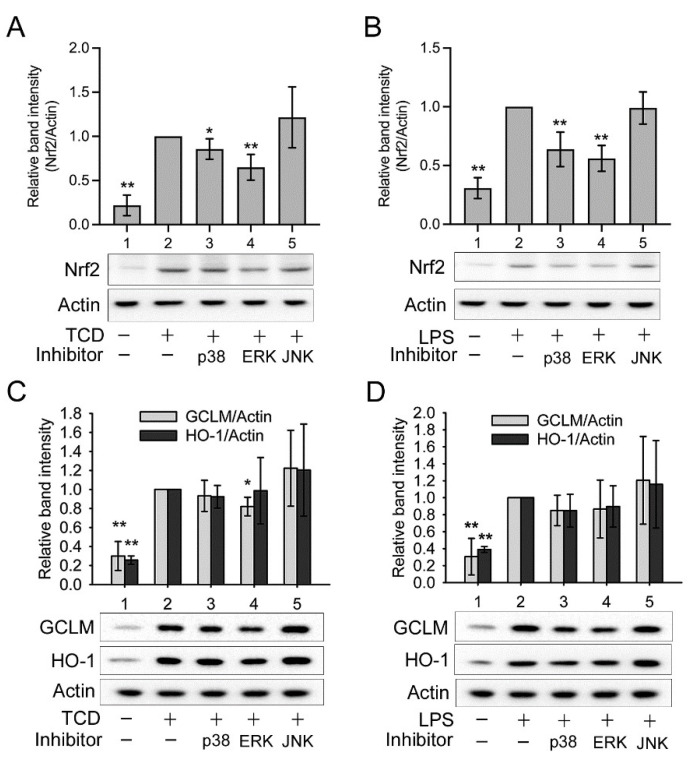
The effects of MAPK inhibitors on TCD- or LPS-activated Nrf2/HO-1 pathway. RAW 264.7 cells were treated with the solvent (Lane 1), 50 μM TCD ((**A**,**C**), Lanes 2–5), or 100 ng/mL LPS ((**B**,**D**), Lanes 2–5) for 2 h (**A**,**B**) or 6 h (**C**,**D**). An inhibitor (20 μM) of p38 (Lane 3), ERK1/2 (Lane 4), or JNK (Lane 5) was also added to cells treated with TCD or LPS. The levels of Nrf2 (**A**,**B**), GCLM (**C**,**D**), and HO-1 (**C**,**D**) were analyzed using Western blotting. Normalized relative band intensity versus Lane 2 was determined. Data are presented as the means ± standard deviations of four independent experiments. * *p* < 0.05, ** *p* < 0.005 versus Lane 2.

Nonetheless, MAPKs also activate cytoprotective and anti-inflammatory factors. They have been shown to activate nuclear factor erythroid 2-related factor 2 (Nrf2), leading to the expression of antioxidant enzymes, including heme oxygenase-1 (HO-1) and the glutamate–cysteine ligase modifier subunit (GCLM) [14,15,16]. Therefore, we further determined whether TCD activated the Nrf2/HO-1 pathway. As displayed in Figure 8D,E, TCD (Lane 2), LPS (Lane 3), and LPS + TCD (Lane 4) all significantly enhanced the expression of HO-1 (D) and GCLM (E) compared with the control (Lane 1). Consistently, these treatments also elevated Nrf2 expression (Figure 8F, Lanes 2–4). These data confirm that TCD and LPS both activated the Nrf2/HO-1 pathway. To further assess the roles of MAPKs, a p38, ERK1/2, or JNK inhibitor was added to cells cotreated with LPS and TCD (Figure 8F; Lanes 5, 6, and 7, respectively). None of the inhibitors significantly blocked Nrf2 activation by LPS and TCD. Similarly, the expression of HO-1 and GCLM induced by LPS + TCD (Figure 8G,H, Lane 4) was not apparently inhibited by any single MAPK inhibitor (Figure 8G,H, Lanes 5–7). Furthermore, the respective effect of TCD or LPS on MAPKs was also examined using the MAPK inhibitors. Figure 9A shows that TCD-promoted Nrf2 expression (Lane 2) was partially yet significantly inhibited by p38 inhibitor (Lane 3) or ERK1/2 inhibitor (Lane 4), but not by JNK inhibitor (Lane 5). Similarly, LPS-elevated Nrf2 expression (Figure 9B, Lane 2) was also partially and significantly suppressed by p38 inhibitor or ERK1/2 inhibitor (Figure 9B, Lanes 3 and 4), but not by JNK inhibitor (Figure 9B, Lane 5). However, Figure 9C,D exhibit that TCD- (C, Lane 2) or LPS-induced (D, Lane 2) GCLM and HO-1 expression was repressed by p38 inhibitor (Lane 3) or ERK inhibitor (Lane 4) in a milder, less apparent extent. In agreement with the results of Figure 9A,B, JNK inhibitor did not inhibit the effect of TCD or LPS on inducing GCLM and HO-1 expression (Figure 9C,D, Lane 5). The results of Figure 9 suggest that JNK is probably not involved in activating the Nrf2/HO-1 pathway.

## 3. Discussion

In this study, chemical isolation from the triterpenoid-rich fractions of the extracts of the vines and leaves of *M. charantia* yielded predominantly four cucurbitane-type triterpenoids: momordicines I, II, and IV, and TCD. Momordicine I exerted deleterious effects on the growth of normal cell lines, momordicine II had a milder adverse effect on cells, and momordicine IV and TCD were not harmful to normal cells at 0.1–100 μM. Therefore, in view of safety, momordicine IV and TCD are more appropriate for potential development in biomedical applications than are momordicine I or II. However, these findings suggest that the extract of vines and leaves from *M. charantia* contains potentially toxic chemicals and thus should be used with caution.

The anti-inflammatory potential of TCD was more notable than those of momordicines II or IV; thus, we further characterized it. To reiterate, TCD was indicated to have anti-inflammatory activity both in vivo and in vitro because it inhibited LPS-induced phagocytosis and the expression of iNOS, NO, TNF-α, and IL-6 in a macrophage cell model. Furthermore, it ameliorated ear edema in an animal model. The underlying mechanism likely hinges on the suppression of the IKK/NF-κB pathway. However, LPS enhanced the expression of Nrf2, HO-1, and GCLM, which may provide protection against excessive inflammatory responses [17], whereas TCD also increased the expression of these proteins whether when used alone or together with LPS. HO-1 reduces cellular oxidative damage and mitigates inflammatory responses [18,19,20]. Thus, TCD likely protected cells from oxidative damage by activating the Nrf2/HO-1 pathway, which also helped reduce inflammation. However, TCD activated MAPKs and did not suppress MAPK activation by LPS. MAPKs increase the expression of some proinflammatory cytokines, which may explain why TCD only partially inhibited LPS-induced production of inflammatory cytokines, as shown in Figure 5E,F.

MAPKs are suggested to activate the Nrf2/HO-1 pathway [14,15,16], and ERK1/2 and p38 have been reported to activate Nrf2 [18,21,22], whereas the role of JNK is less clear. TCD and LPS both activated p38, ERK1/2, and JNK. Our data suggest that p38 and ERK1/2 are both involved in LPS- or TCD-induced activation of Nrf2, whereas JNK is not. TCD- or LPS-promoted Nrf2 expression was both partially yet significantly inhibited by either p38 inhibitor or ERK inhibitor, though the suppressive effects of these inhibitors declined on the Nrf2 downstream factors HO-1 and GCLM. Theoretically, if p38 and ERK1/2 are both involved in activating Nrf2, when one of these kinases is inhibited, Nrf2 can still be activated by the other one. Therefore, the observed partial decrease of Nrf2 level by either p38 or ERK1/2 inhibitor is reasonable. In TCD and LPS-cotreated cells (Figure 8F–H), LPS and TCD may synergistically activate p38 or ERK1/2 when one of these kinases is inhibited, resulting in a higher extent of Nrf2 activation. Consequently, significant Nrf2 suppression by either inhibitor could not be observed in Figure 8F.

**Figure 10:**
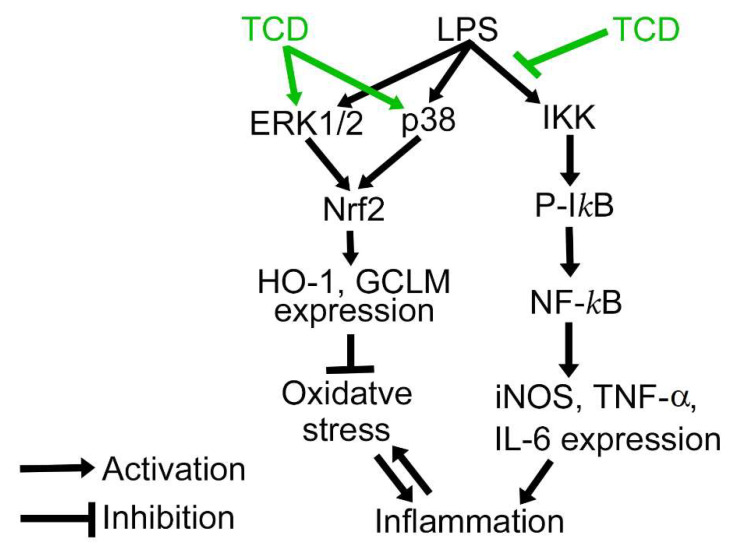
The proposed anti-inflammatory mechanism of TCD. TCD inhibits the LPS-induced activation of IKK. Moreover, TCD, together with LPS, activates ERK1/2 and p38, resulting in elevated expression of Nrf2 and the antioxidant enzymes HO-1 and GCLM to protect cells from oxidative damage. P-IκB, phosphorylated IκB.

On the basis of our findings, we propose the anti-inflammatory mechanism of TCD (Figure 10). TCD can reduce LPS-induced inflammation through inhibiting IKK activation by LPS. Moreover, TCD and LPS both activate ERK1/2 and p38. These kinases activate Nrf2 and increase the expression of the antioxidant enzymes HO-1 and GCLM. Inflammation can exacerbate oxidative stress; conversely, oxidative stress can aggravate inflammation. Antioxidant enzymes can protect cells from oxidative damage and thus prevent excessive amounts of inflammation. In other words, TCD reduces the extent of inflammation and protects cells from oxidative damage.

Momordicines II and IV are the glucosides of momordicine I. In cytotoxicity assays, the GI_50_ of momordicine I was lower than that of momordicine II in IEC-18 cells. Moreover, momordicine I was lethal to FL83B cells and RAW 264.7 cells in concentrations higher than 10 μM, whereas 20–100 μM momordicine II did not inhibit the growth of these cells. Momordicine IV exhibited no harmful effect on any of the three tested cell lines. Thus, the order of cytotoxicity is momordicine I > momordicine II > momordicine IV. This suggests that glycosylation reduces the cytotoxicity of momordicine I, and that glycosylation on the –OH group of C-7 (momordicine IV) reduces toxicity to a greater extent than does glycosylation on the –OH group of C-23 (momordicine II).

TCD and momordicine I are isomers with similar structures, but their cytotoxic effects differ substantially. TCD exerts considerably less deleterious effects on cells than does momordicine I. These two isomers differ structurally between C-23 and C-25. The –OH group on C-25 of TCD is shifted to C-23 in momordicine I; the double bond between C-23 and C-24 in TCD is moved to between C-24 and C-25. This structural rearrangement notably affects the cytotoxicity. Future studies should investigate the cellular targets that interact with momordicine I and cause the cytotoxic effect.

Although cytotoxic, momordicine I exhibited a potential anti-inflammatory effect in that it reduced the LPS-induced iNOS expression at its sublethal concentration (10 μM). Thus, momordicine I and TCD both likely contribute to the anti-inflammatory activity of the extract of vines and leaves from *M. charantia*. However, momordicine I may also contribute to the adverse effects of the extract.

High lipophilicity may facilitate the permeation of a compound through biomembranes, resulting in higher bioactivity or cytotoxicity of the compound [23,24,25]. The lipophilicity of the compounds reported here is TCD > momordicine I > momordicine II = momordicine IV according to their logP values. This lipophilicity order may explain why TCD and momordicine I are more bioactive than momordicine II and momordicine IV, but it cannot explain why momordicine I is the most cytotoxic among them. Moreover, momordicine I exhibited a potential anti-inflammatory effect at its sublethal concentration, suggesting that momordicine I may be as effective as or even better than TCD in anti-inflammation. Therefore, in addition to lipophilicity, there are likely other factors that influence the bioactivity or cytotoxicity of these compounds. For example, MAPKs regulate mitochondrial metabolism [26], and some compounds were reported to target mitochondria for their cytotoxic or anti-inflammatory effects [24,27,28]. Thus, whether the triterpenoids reported in this study target mitochondria deserves to be addressed. Moreover, some lipophilic compounds including nobiletin, kaempferol, luteolin, quercetin, and tangeretin were suggested to enter cells through an energy independent carrier-mediated system [29]. It cannot be excluded that such a system also exists for the compounds reported here.

TCD was also found in the fruits of *M. charantia* L. [30]. This compound has been reported to reduce blood glucose levels in diabetic animal models [30] and serve as an insulin sensitizer and insulin substitute [31]. Our findings further demonstrate that it is an anti-inflammatory agent. Moreover, TCD was also isolated from the leaves of wild varieties of *M. charantia* previously [32] and was demonstrated to reduce periodontal pathogen- or *Cutibacterium acnes*-induced inflammatory responses in human monocytic THP-1 cells in vitro and ameliorate inflammation in the corresponding pathogen-stimulated mouse models in vivo [32,33]. Therefore, studies from different groups all support that TCD is an anti-inflammatory agent. In sum, TCD, which is distributed in the fruits, vines, and leaves of *M. charantia* L., has hypoglycemic effects and anti-inflammatory functions. It can be regarded as a potential new agent for developing therapeutic or health-care products of anti-inflammation or glycemic control, or as an index ingredient for the bioactivity of bitter melon extracts.

Our results indicate that although momordicine I is cytotoxic, TCD is relatively safe and exerts notable anti-inflammatory effects. Therefore, a protocol that can enrich the extraction of TCD from the vines and leaves of *M. charantia* but remove momordicine I would likely enhance the anti-inflammatory effect and improve the safety of the extract.

## 4. Materials and Methods

### 4.1. Chemicals and Reagents

Antibodies for Nrf2, phosphorylated IKK, IKK-α, IKK-β, phosphorylated IκB, p65, phosphorylated JNK, total JNK, phosphorylated p38, total p38, phosphorylated ERK1/2, total ERK1/2, HO-1, an FITC-conjugated secondary antibody, and U0126 (ERK1/2 inhibitor) were purchased from Cell Signaling Technology (Beverley, MA, USA). A GCLM antibody and SB202190 (p38 inhibitor) were ordered from Abcam (Cambridge, UK), an iNOS antibody was supplied by BD Biosciences (Franklin Lakes, CA, USA), an actin antibody was purchased from Millipore (Bedford, MA, USA), and horseradish peroxidase–conjugated secondary antibodies were provided by Santa Cruz Biotechnologies (Santa Cruz, CA, USA). A protease inhibitor cocktail, a phosphatase inhibitor cocktail, and SP600125 (a JNK inhibitor) were supplied by Calbiochem (Merck Millipore, Darmstadt, Germany). Cell Culture Lysis Reagent was purchased from Promega (Madison, WI, USA). Bradford reagent was purchased from Bio-Rad (Hercules, CA, USA). Fetal bovine serum was acquired from Invitrogen (Carlsbad, CA, USA). Cell culture media, dimethylsulfoxide (DMSO), and other chemicals were ordered from Sigma Chemical Company (St. Louis, MO, USA).

### 4.2. General Experimental Procedures

The NMR spectra were obtained at 400 and 100 MHz for ^1^H and ^13^C, respectively, on a Varian MR 400 NMR spectrometer (Agilent Technologies, Santa Clara, CA, USA) in CDCl_3_ or pyridine-*d*_5_, with solvent peaks as references. Silica gel 60 (Merck Millipore, 230–400 mesh), MCI gel CHP20P (Merck Millipore, 75–150 μm), and LiChroprep RP-18 (Merck Millipore, 40–63 μm) were used for column chromatography. Precoated silica gel plates (Merck Millipore, Kieselgel 60 F_254_, 0.25 mm) and precoated RP-18 F_254s_ plates (Merck Millipore, 1.05560) were employed in TLC analyses. Spots were visualized under ultraviolet (UV) light or heating silica gel plates sprayed with 10% H_2_SO_4_ in CH_3_OH. High-performance liquid chromatography (HPLC) was performed on a Primaide 1110 pump equipped with a Primaide 1410 UV detector at 220 nm and a semipreparative reversed-phase column (Merck Millipore, Hibar Purospher RP-18e, 5 μm, 250 mm × 10 mm).

### 4.3. Plant Materials

In July 2018, vines and leaves of *M. charantia* were collected from a local farm in Kenting, Pingtung County, Taiwan. A voucher specimen (MC-SL-2018) was authenticated by Prof. Sheng-Zehn Yang, curator of the herbarium of the Department of Forestry at National Pingtung University of Science and Technology and kept in the Department of Life Sciences at National University of Kaohsiung.

### 4.4. Extraction and Isolation of Triterpenoids

The air-dried vines and leaves (1.0 kg dry wt.) of *M. charantia* were ground and extracted three times with methanol (MeOH; 3 × 1.5 L, 3 days each) in a percolator at room temperature. The combined extracts were filtered and concentrated under vacuum to obtain a crude extract (52.0 g). The extract was stirred twice with H_2_O (2 × 1.5 L), and the resulting emulsion was separated from the residue and partitioned between EtOAc-H_2_O (1:1) to obtain an EtOAc extract (35.0 g). This extract was fractionated on a silica gel column by elution with *n*-hexane, *n*-hexane−EtOAc, and EtOAc−MeOH mixtures of increasing polarity to obtain 29 fractions on the basis of TLC. Next, ^1^H NMR spectroscopy data were examined to identify the triterpenoid-rich fractions. Among the fractions containing cucurbitane-type triterpenoids, fractions 19 and 27 were of further interest. Fraction 19 (5.0 g) eluted with *n*-hexane−EtOAc (1:8) was subjected to column chromatography on RP-18 gel by elution with MeOH−H_2_O mixtures of increasing polarity to obtain two subfractions. The subfraction 19-1 (4.0 g) eluted with 80% MeOH in H_2_O was further loaded onto an RP-18 gravity column and eluted with 60% MeOH in H_2_O, 70% MeOH in H_2_O, 80% MeOH in H_2_O, 90% MeOH in H_2_O, and 100% MeOH. Five subfractions were obtained, of which subfraction 19-1-3 (3.30 g) was purified using RP-18 HPLC (80% MeOH in H_2_O) to obtain TCD (0.78 g) and momordicine I (1.06 g). Fraction 27 (6.0 g) eluted with EtOAc−MeOH (1:1) was loaded onto an MCI gel column and eluted with a MeOH−H_2_O gradient (70:30 to 100% MeOH) to obtain 10 subfractions. The subfraction 27-2 (1.0 g) eluted with 60% MeOH in H_2_O was further loaded onto an RP-18 gravity column (MeOH/H_2_O, 60:40 to 100% MeOH) for separation into eight subfractions. Subsequently, subfraction 27-2-5 (568 mg) was purified using RP-18 HPLC (77% MeOH in H_2_O) to obtain momordicine II (76.0 mg) and momordicine IV (75.0 mg). The purity of each compound was >99% according to their HPLC profiles.

### 4.5. Cell Culture and Cytotoxicity Assays

IEC-18, FL83B, and RAW 264.7 cells were purchased from the Bioresource Collection and Research Center (Hsinchu, Taiwan) and cultured at 37 °C in a humidified incubator supplied with 5% CO_2_, using the medium specified in the supplier’s instructions.

Cells were seeded in 96-well plates and treated in triplicate with the desired compound at the indicated concentration (see Section 2.2) or an equal volume of the solvent (control; DMSO for the triterpenoids; phosphate-buffered saline (PBS; pH 7.4) for LPS) in serum-free medium for 24 h. A Quick Cell Proliferation Colorimetric Assay Kit (BioVision, Milpitas, CA, USA) was then employed to analyze the relative cell numbers, as described previously [34]. The survival rate relative to the control was determined, and the mean ± standard deviation was calculated.

### 4.6. Western Blotting

RAW 264.7 cells were seeded in 35-mm or 60-mm plates and subjected to chemical treatment as described in Section 2. The cells were then washed twice with PBS, submerged in a lysis buffer (Cell Culture Lysis Reagent containing a protease inhibitor cocktail and a phosphatase inhibitor cocktail when phosphorylated proteins were the targets of analysis), and scraped off the plate placed on ice. The resulting suspension was centrifuged at 12,000× *g* for 10 min at 4 °C. The supernatant was collected and analyzed for the protein concentration by using Bradford reagent. Equal amounts of protein were sampled from different treatments and subjected to electrophoresis and Western blotting, as described previously [34,35]. Immunoreactive bands were detected using the UVP BioSpectrum imaging system (Upland, CA, USA). The band intensities were determined using the supplied software.

### 4.7. Phagocytosis Analysis

RAW 264.7 cells were seeded in 96-well plates and subjected to chemical treatment (as described in Figure 5) for 24 h at 37 °C. After being washed twice with PBS, cells were incubated in PBS containing 40 μg/mL neutral red for 1 h at 37 °C. Next, cells were washed twice with PBS and incubated in a lysis solution (acetic acid:ethanol = 1:1, *v*/*v*) for 1 h at room temperature [36]. Subsequently, absorbance at 492 nm was detected using a microplate reader (Spectramax Plus 384, Molecular Devices, San Jose, CA, USA).

### 4.8. Analysis of NO and Cytokines

The concentration of NO in the culture medium was analyzed using a Griess reagent kit according to the manufacturer’s protocol (Promega). TNF-α and IL-6 concentrations in the culture medium were analyzed using the respective ELISA kits in compliance with the manufacturer’s instructions (eBioscience, Thermo Fischer Scientific, Vantaa, Finland).

### 4.9. Animal Experiments

All animal experiment protocols were approved by the Institutional Animal Care and Use Committee of National Pingtung University of Science and Technology (approval no. NPUST-105-088). The mouse ear edema assay was conducted as described previously [37] with some modifications. In brief, after 2 weeks of acclimation, 8-week-old male ICR mice weighing 30–35 g (BioLASCO, Taipei, Taiwan) were divided into six groups of six. TPA (3 μg/ear dissolved in 20 μL of acetone) was applied on the left ears of the mice in Groups 2–6, whereas 20 μL of acetone was applied to the left ears of the mice in Group 1. One hour later, 500 μg/ear indomethacin (dissolved in 20 μL of acetone) was applied to the left ears of the mice in Group 3, whereas 250, 500, and 750 μg/ear TCD (dissolved in 20 μL butanol) was applied to the left ears of the mice in Groups 4, 5, and 6, respectively. To the left ears of the mice in Groups 1 and 2, 20 μL of butanol was applied. The thickness of the treated ears was measured using a dial thickness gauge (Peacock, Ozaki, Tokyo, Japan) before and 4, 16, and 24 h after TPA stimulation.

### 4.10. Confocal Microscopy

Confocal microscopy was performed as described previously [31]. In brief, RAW 264.7 cells were grown on 18 mm × 18 mm glass coverslips pretreated with 1% gelatin A and placed in 35-mm dishes. After treatment, as described in Section 2, the cells were fixed, permeabilized, and blocked with 1% BSA as described previously [31]. The cells were then incubated overnight with an anti-p65 antibody at 4 °C. After being thoroughly washed with 1% BSA, the cells were incubated with an FITC-conjugated secondary antibody at room temperature for 1 h in the dark and then washed with 1% BSA. Subsequently, the cells were stained with rhodamine phalloidin for 30 min, washed with 1% BSA, and stained with 5 μg/mL DAPI in PBS for 3 min. After being washed with 1% BSA, the cells were mounted in mounting medium (Vector Laboratories, Burlingame, CA, USA) onto glass slides. The intracellular location of p65 was then examined using a confocal microscope (FV1000, Olympus, Tokyo, Japan). The images were processed using the supplied software (Olympus Fluoview Ver.4.2b).

### 4.11. Statistical Analysis

Data were analyzed using one-way analysis of variance followed by Scheffe’s post hoc test. Differences were considered significant at *p* < 0.05 and *F* > 3.5546.

### 4.12. LogP Calculation

The LogP value of each compound was calculated using a free App (ALOGPS 2.1 program provided by Virtual Computational Chemistry Laboratory; http://www.vcclab.org; accessed on 1 October 2021).

## Data Availability

All data are reported in the manuscript and in the Supplementary Material.

## Supplementary Materials

The following are available online at https://www.mdpi.com/article/10.3390/ijms23031071/s1. Figure S1: the NMR spectra of momordicines I, II, and IV, and logP calculation for TCD, momordicines I, II, and IV.
