# Supplementary material for: Cytotoxic and Anti-Inflammatory Triterpenoids in the Vines and Leaves of Momordica charantia
Source: Int J Mol Sci. 2022 Jan 19;23(3):1071. doi: 10.3390/ijms23031071 (PMC8834831; doi:10.3390/ijms23031071)
Supplement: Supplementary file 1 [file ijms-23-01071-s001.zip › Figure S1.pdf]

^{1}H (400 MHz) NMR spectra of momordicine I ($CDCl_3$)

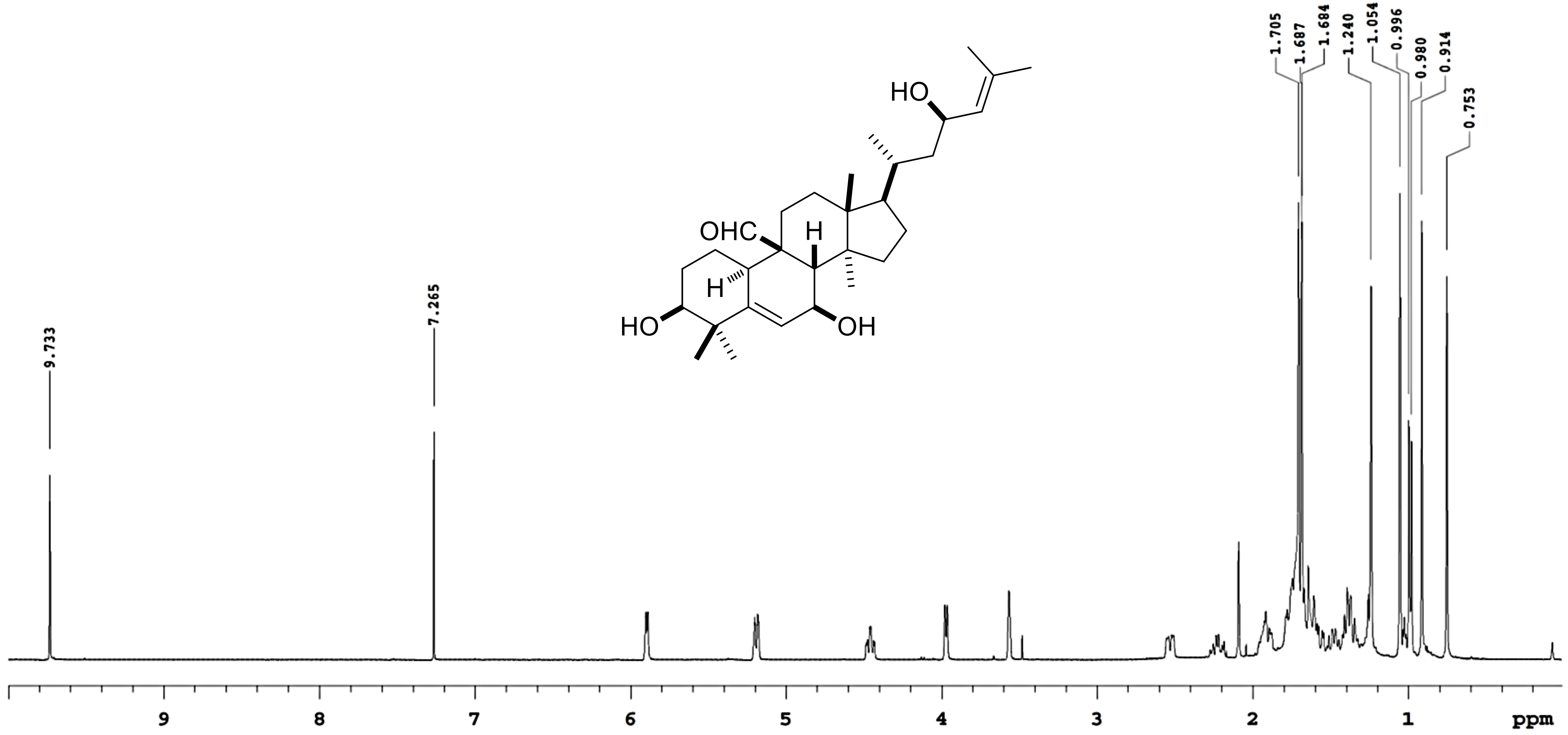

^{13}C (100 MHz) NMR spectra of momordicine I ($CDCl_3$)

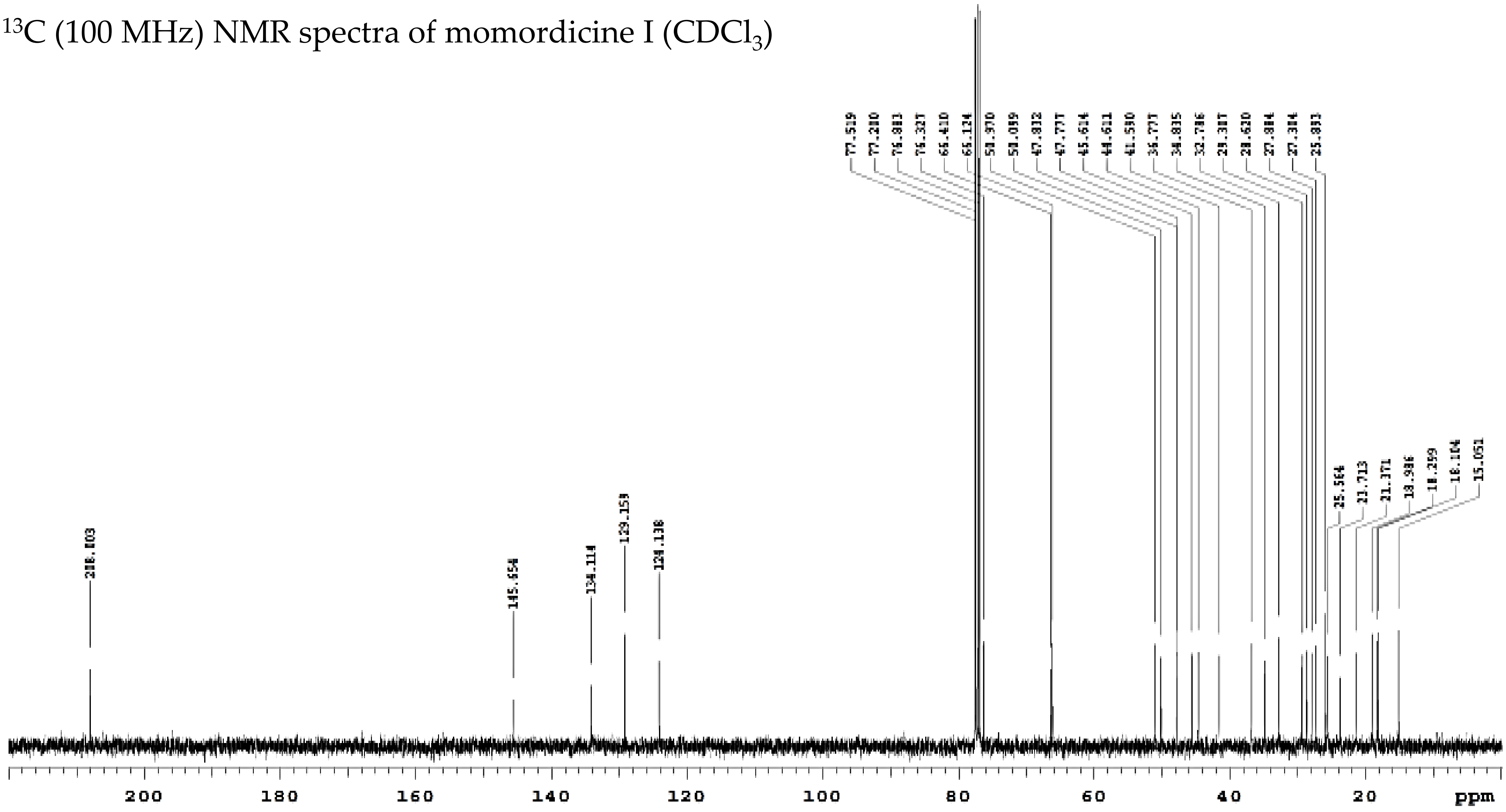

^{1}H (400 MHz) NMR spectra of momordicine II (pyridine-d_5)

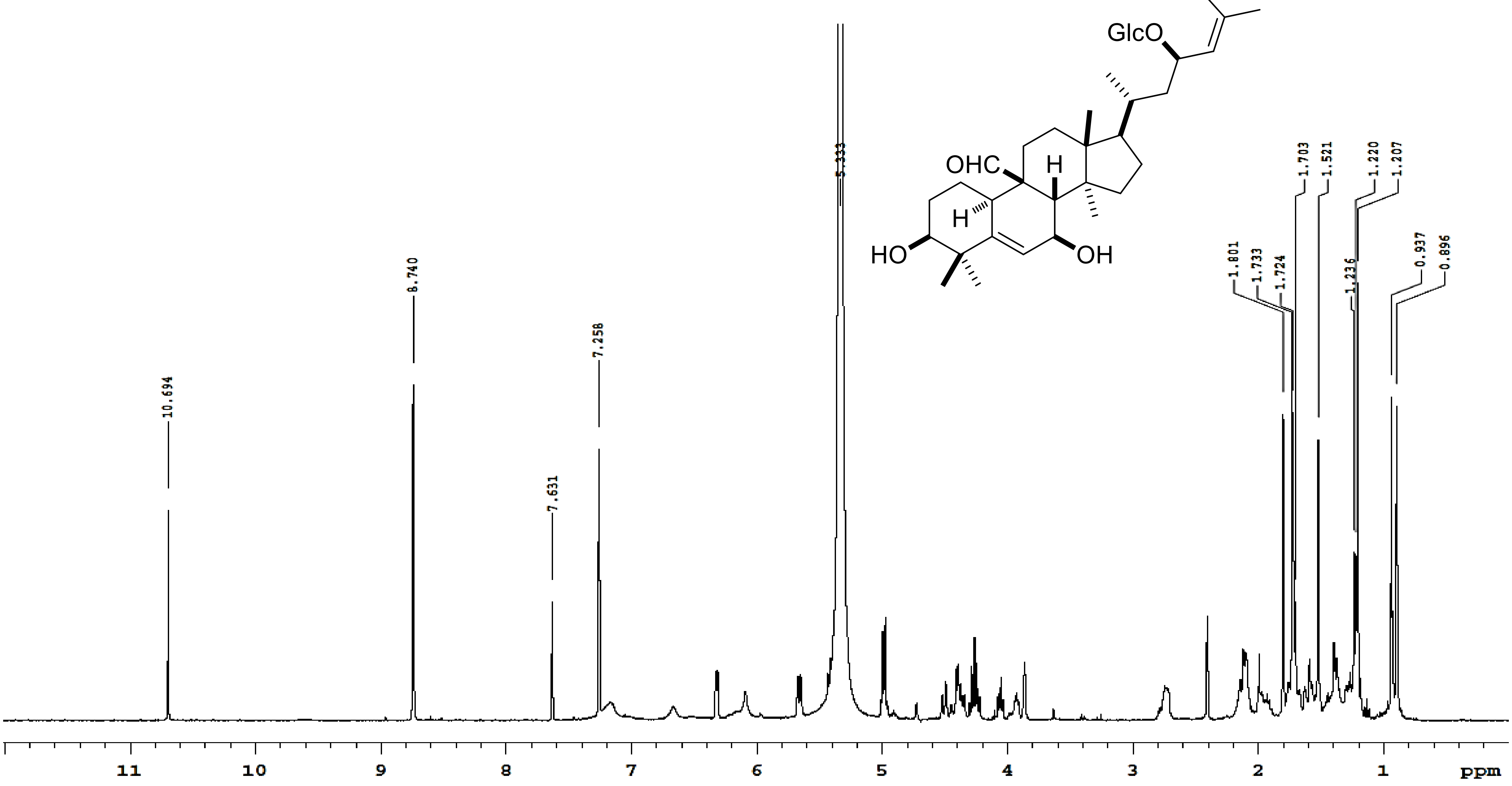

^{13}C (100 MHz) NMR spectra of momordicine II (pyridine-d_5)

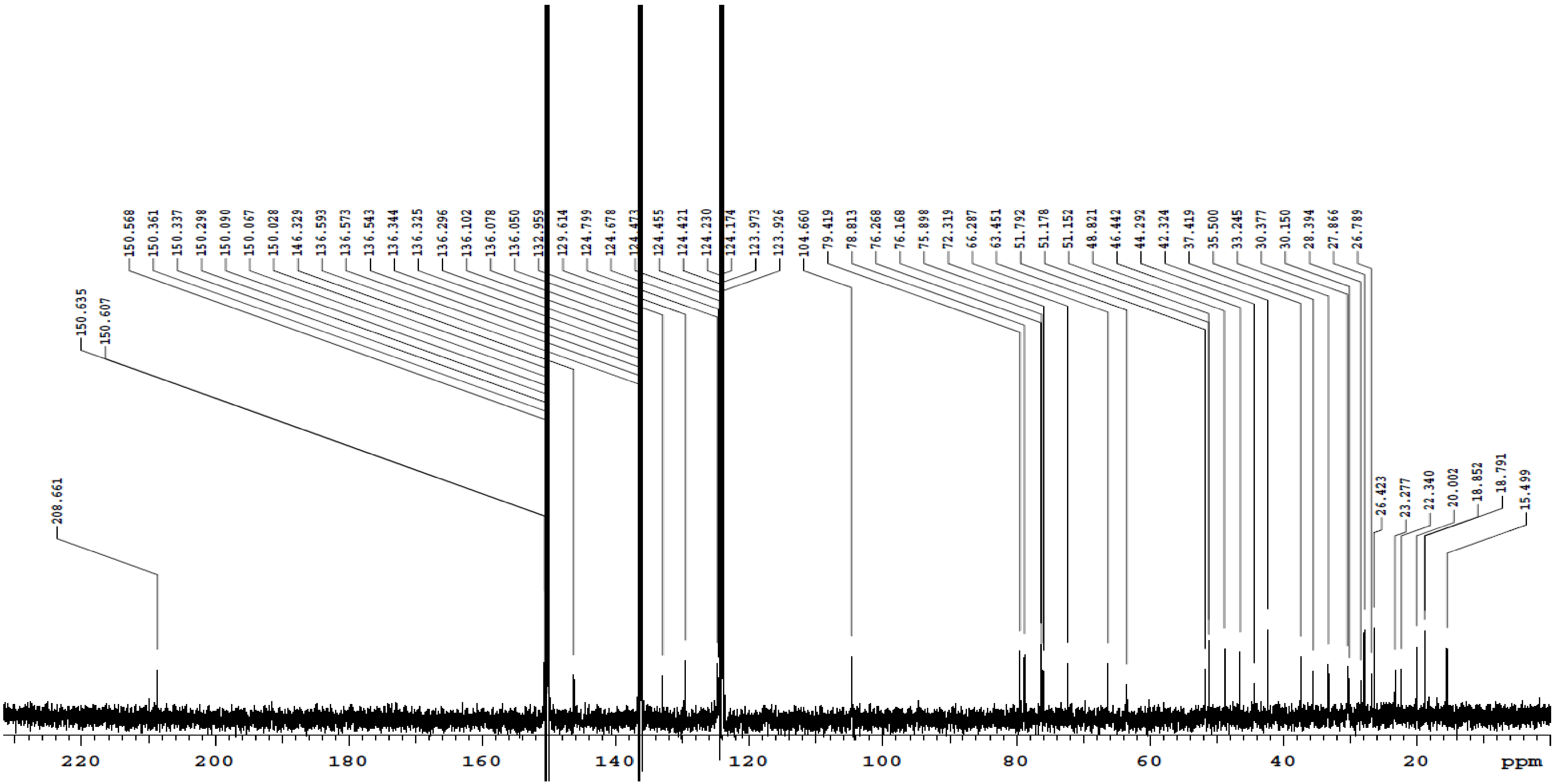

^{1}H (400 MHz) NMR spectra of momordicine IV (pyridine-d_5)

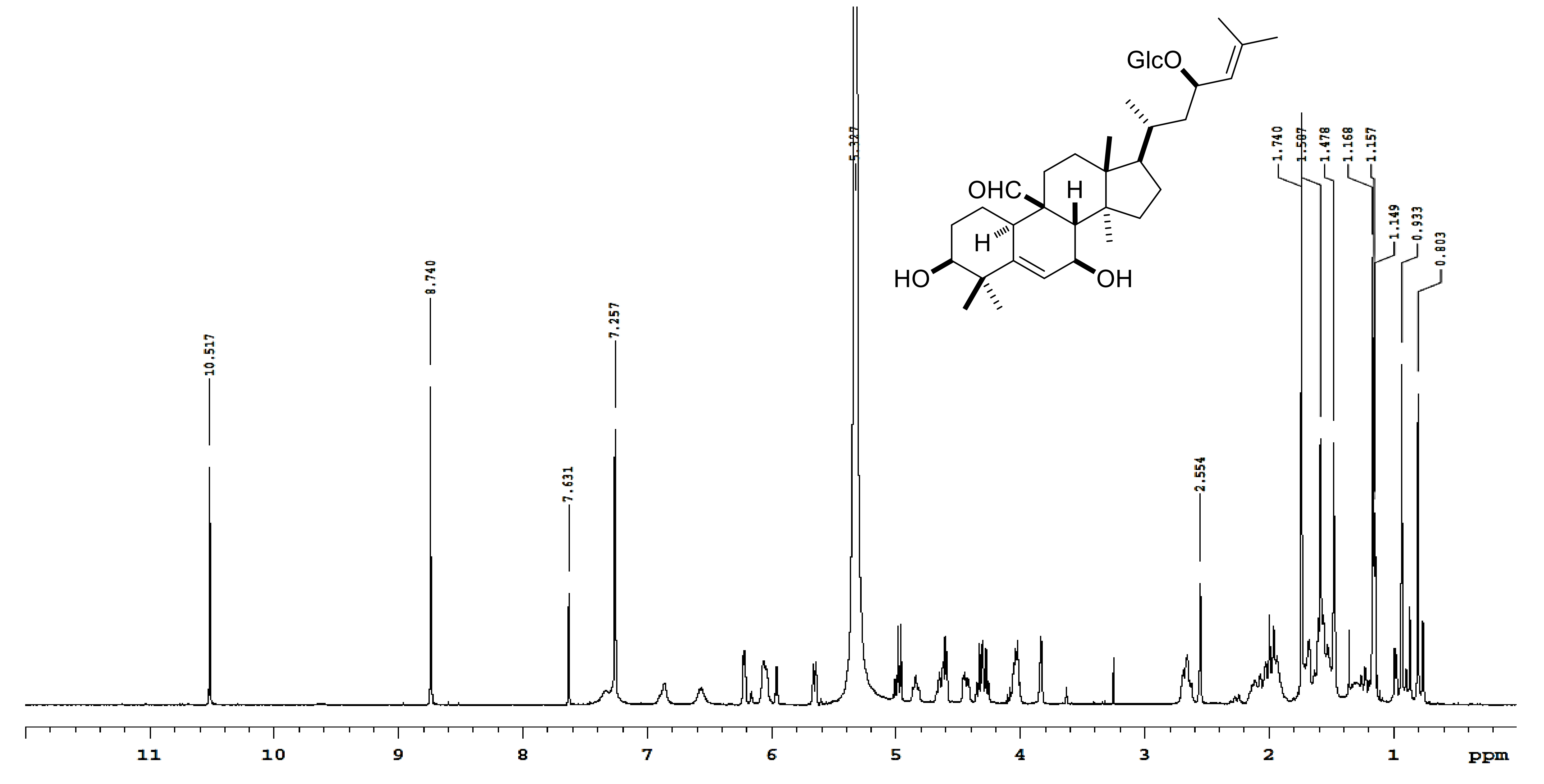

^{13}C (100 MHz) NMR spectra of momordicine IV (pyridine-d_5)

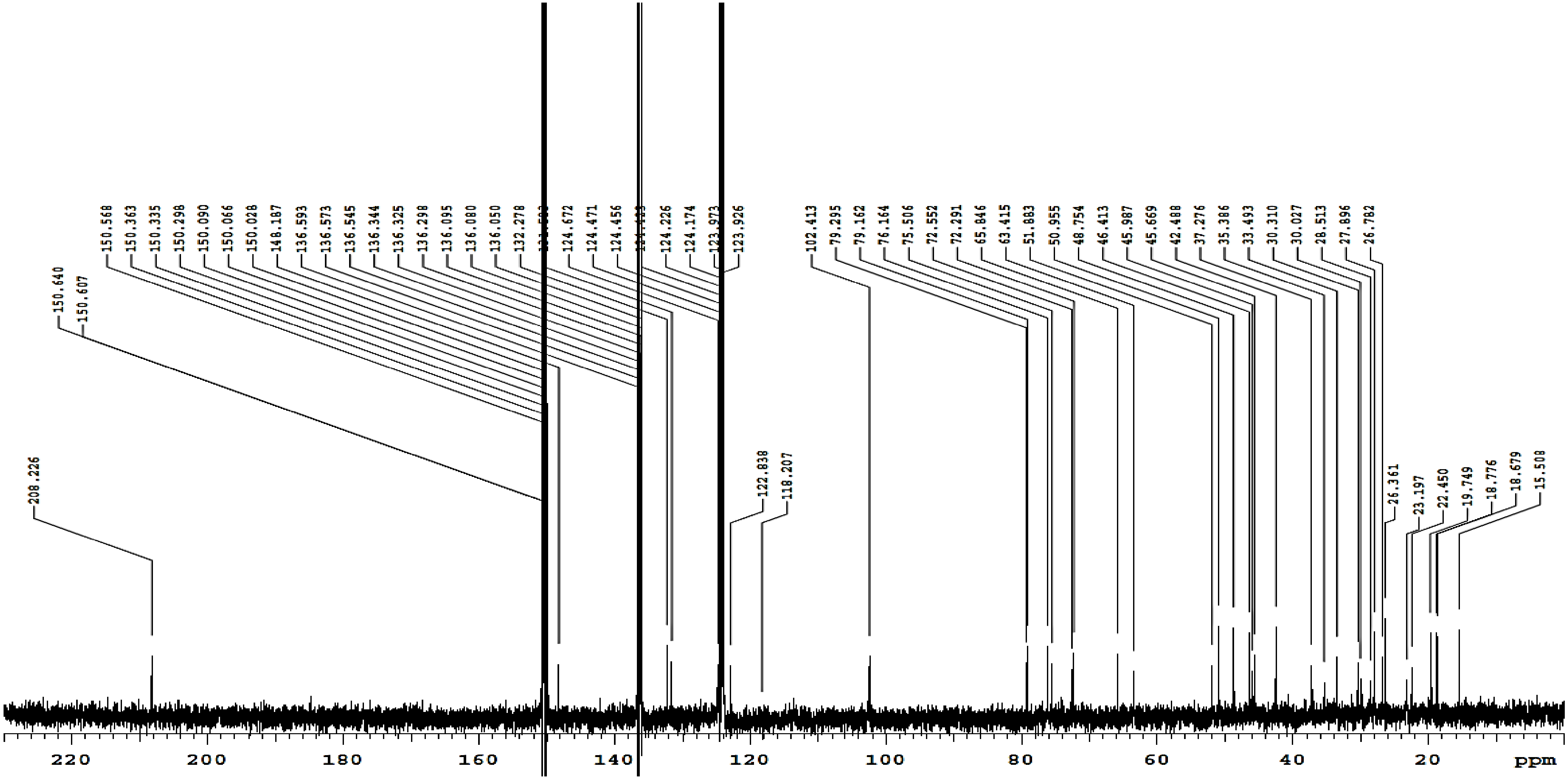

LogP calculation for TCD and momordicine I

TCD

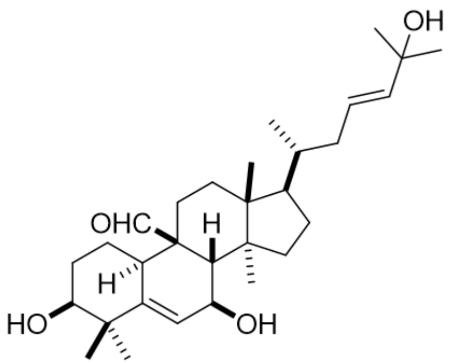

mol_N logP logS SMILES
mol_1 5.17 -5.12 OC(C(C)(C)C1=C2)CCC1C3(C=O)C(C(CCC4C(CC=CC(C)(O)C)C)(C)C4(C)CC3)C2O

Momordicine I

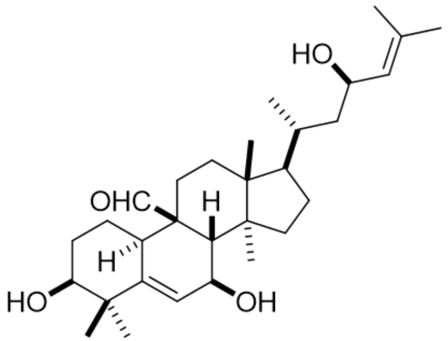

mol_N logP logS SMILES
mol_1 4.41 -4.81 CC12C(CCC2C(CC(C=C(C)C)O)C)(C)C3C(O)C=C4C(C)(C)C(O)CCC4C3(C=O)CC1

LogP calculation for momordicines II and IV

Momordicine II

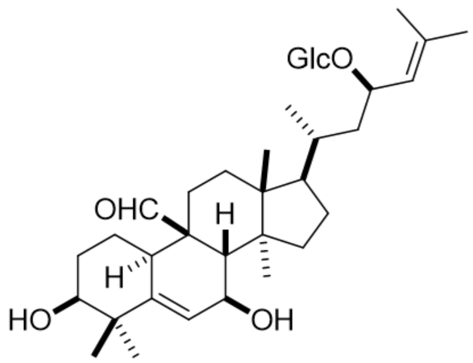

mol_N logP logS SMILES
mol_1 2.72 -4.23 CC12C(CCC2C(CC(OC(C3O)OC(CO)C(O)C3O)C=C(C)C)C)(C)C4C(O)C=C5C(C)(C)C(O)CCC5C4(C=O)CC1

Momordicine IV

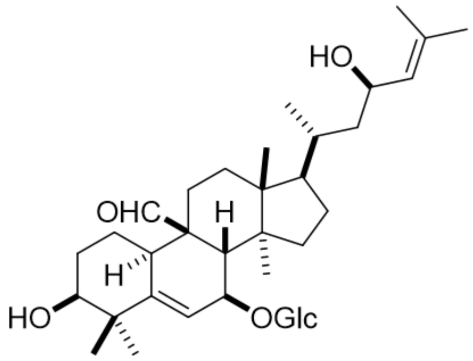

mol_N logP logS SMILES
mol_1 2.72 -4.04 CC12C(CCC2C(CC(C=C(C)C)O)C)(C)C3C(OC(C4O)OC(CO)C(O)C4O)C=C5C(C)(C)C(O)CCC5C3(C=O)CC1
